# US Gulf Coast states: The rise of neglected tropical diseases in "flyover nation"

**DOI:** 10.1371/journal.pntd.0005744

**Published:** 2017-11-16

**Authors:** Peter J. Hotez, Sheila Jackson Lee

**Affiliations:** 1 National School of Tropical Medicine and Texas Children’s Hospital Center for Vaccine Development at Baylor College of Medicine, Houston, Texas, United States of America; 2 United States House of Representatives for the Eighteenth Congressional District of Texas, Houston, Texas, United States of America; University of Melbourne, AUSTRALIA

In 2016, as Zika virus infection spread north from Brazil into Venezuela, Colombia, Central America, and the Caribbean, there were concerns that it would emerge in states on the United States Gulf Coast, especially in the urban centers of South Texas and Florida. The vulnerability of this region to Zika stemmed from the presence of recent and previous arbovirus outbreaks during the 2000s in South Texas and Florida, together with a persistent warm and subtropical climatic region with significant poverty. New mapping data indicate that population densities of the mosquito *Aedes aegypti*, the leading arthropod vector transmitting this virus, are highest on the Gulf Coast relative to other regions of the continental US [1]. Despite the possibility of an imminent threat of Zika virus, transmission on the Gulf Coast, and significant risks to pregnant women living in poverty, US Congressional action through emergency funding from supplemental appropriations for state and local health authorities was delayed until the fall of 2016—well past the summer, when arbovirus transmission historically is at its peak on the Gulf Coast. Zika virus transmission ensued in both South Texas and Florida, continuing through the winter months in the former.

Some critics of the US Government’s lack of a timely response suggested in public testimony before congressional committees and in the media that the US Government was slow in acting because Zika mostly threatened the urban disadvantaged people living on the Gulf, a neglected population living in a “flyover nation.” This term refers to the interior of the nation ignored by a government and media biased in favor of urban centers in the Northeast, Southern California, or Silicon Valley [2].

Further analysis reveals that the US Gulf Coast is not only vulnerable to Zika but also to a range of neglected tropical diseases (NTDs). NTDs are illnesses that impact disproportionately in impoverished populations and actually perpetuate poverty because of chronic, deleterious effects on wage-earning and productivity and maternal and child health and development.

Shown in Table 1 are new estimates for the most common NTDs now pervasive in the 5 US Gulf states—Texas, Louisiana, Mississippi, Alabama, and Florida—states composed of communities with the current and historically highest rates of poverty in America in a region most affected by economic declines and environmental, climate, and industrial disasters [3–12].

**Table 1:** Prevalence and incidence estimates of the NTDs affecting the US Gulf Coast states.

Disease	Estimated Cases in the Gulf Coast States	Cases Nationally	Comments
Toxocariasis	>2.2 million prevalent cases	Not determined	Based on 21% [3] of African American population of US Gulf Coast states (10,758,201) in 2015 [4]
Trichomoniasis	1.4 million	Not determined	Based on the means of 4.5% and 4.7% for Texas and Florida, respectively [5] for women (30 million) extrapolated across the US Gulf Coast states [4]
Chagas disease	58,050	24.4%	[6]
West Nile virus infection	8,864 reported cases 1999–2016, including 1,868 cases in Texas 2012	19.2% of incident cases between 1999 and 2016; 45.6% of incident cases in 2012	[7]
Typhus group rickettsiosis	1,762 cases 2003–2013	Approximately100%	[8]
Neurocysticercosis	242–937	18.5%	Based on 1.5–5.8 cases per 100,000 Hispanics [9] (16,152,682 Hispanic population in Gulf Coast states in 2015, according to [4])
Hansen's disease	565	31.0%	National Hansen’s disease HRSA 10-year cumulative report (2005–2014) of US cases by reporting [10]
Zika virus infection	225 (autochthonous transmission)	100%	[11]
Dengue and chikungunya virus infections	Emerging autochthonous transmission	100%	[12]
Total	3.7 million	Not determined	Determined by adding the numbers in column 2

Abbreviation: HRSA, Health Resources and Services Administration; NTDs, neglected tropical diseases.

Parasitic and vector-borne diseases are believed to be widespread on the US Gulf Coast, but the true prevalence and incidence estimates of these conditions should be considered preliminary and based on minimally available data. For example, the Centers for Disease Control and Prevention (CDC) found through national surveys that approximately 1 in 5 African Americans is seropositive for toxocariasis, a zoonosis caused by *Toxocara canis* and *T*. *cati*, with people living in poverty in the southern US at greatest risk. Similarly, trichomoniasis exhibits its highest prevalence among African American women living in poverty, while neurocysticercosis is found mostly among Hispanics in poverty. Chagas disease has emerged as an important illness because of immigration across our southern border, but recent studies also indicate a significant level of autochthonous transmission of both Chagas disease and typhus in Texas.

Texas is also the epicenter of mosquito-borne diseases. The largest American outbreaks of West Nile virus infection occurred here in 2012, as well as dengue outbreaks in the early 2000s. In addition, the Zika virus emerged in South Texas and Florida in 2016.

Overall, of the 60 million people living in US Gulf Coast states, 3–4 million people (or 5%–6% of the population) are affected by at least 1 NTD. The reasons why this region is susceptible to NTDs require further research and investigation, but empirical data suggest that the disproportionate impact of poverty and new 21st century environmental changes may be significant drivers.

Regarding the impact of poverty, converging data from both the World Health Organization (WHO) and the Global Burden of Disease Study now indicate that most of the world’s poverty-related neglected diseases and NTDs are found among poor people living in wealthy countries, a concept sometimes referred to as “blue marble health” [13]. Approximately 10 million people live below the poverty line on the US Gulf Coast, while more than one-third of people from US counties living in “persistent poverty” (meaning more than 20% of the population lives in poverty) are in Gulf Coast states [14]. For NTDs, poverty equates to substandard housing that exposes residents to insect vectors, a lack of access to sanitation and water, and degraded environments associated with car and truck tire dumping and other hazards relating to mosquito breeding in poor urban areas.

Poverty does not occur in isolation but, instead, operates in concert with marked environmental shifts linked to climate change [14], now affecting the Gulf Coast more than any other region of the continental US. These environmental shifts include diminished freezing events, longer periods of intense heat, and overall increased temperatures, together with rising sea levels, flooding, and intense and frequent extreme weather events, especially hurricanes [14].

The Gulf Coast is still feeling the effects of the British Petroleum (BP) oil disaster, and it is believed that these factors have profoundly influenced human migrations in the region [14]. Nobel laureate and former US Vice President Al Gore noted how climate change and global warming affect the poor first and foremost.

Relevant to NTDs, changing rainfall patterns, flooding, and warmer temperatures are promoting the emergence of both parasitic infections and arbovirus infections such as Zika, dengue, and chikungunya. Also, there are concerns about the possible return of yellow fever. Fueling this emergence is the recent widening of the Panama Canal, which is expected to enable an expansion of shipping traffic to the Gulf Coast and to usher in the periodic, but regular, introduction and spread of new NTDs. A possible example is the emergence of the rat lungworm *Angiostrongylus cantonensis* in nonindigenous Gulf Coast snails, which can result in human eosinophilic meningitis [15].

Together, the severe poverty, climate changes and warming, human migrations, and changing patterns of global shipping produce a “perfect storm” for ongoing NTD transmission on the Gulf Coast. The emergence of Zika virus is just the latest and most publicized example. Accordingly, there is an urgent need for concerted efforts to detect, treat, and manage these infections.

Among the proposed action items are the establishment of a US government interagency task force to confirm the prevalence and incidence of NTDs on the Gulf Coast, the level of autochthonous transmission, and programs of active surveillance to determine the emergence of NTDs. In addition, academic centers of excellence should be created to promote the development of improved diagnostics, drugs, and vaccines for these conditions. We also need economic assessments of their financial impact and role in trapping Gulf Coast residents in poverty. Today, NTDs represent some of the most important and outstanding health disparities in the US. With sufficient political commitment at the federal, state, and local level, we can make a dramatic impact on suffering and poverty.
